# Muscular effort increases hand-blink reflex magnitude

**DOI:** 10.1016/j.neulet.2018.11.046

**Published:** 2019-05-29

**Authors:** R.J. Bufacchi, S. Ponticelli, G. Novembre, M. Kilintari, Y. Guo, G.D. Iannetti

**Affiliations:** a Department of Neuroscience, Physiology and Pharmacology, University College London (UCL), London, UK; b Centre for Mathematics and Physics in the Life Sciences and EXperimental biology (CoMPLEX), University College London, London, UK; c Neuroscience and Behaviour Laboratory, Istituto Italiano di Tecnologia, Rome, Italy

**Keywords:** Defence, Muscular effort, Blink reflex, Threat, Motor system

## Abstract

Defensive motor responses elicited by sudden environmental stimuli are finely modulated by their behavioural relevance to maximise the organism’s survival. One such response, the blink reflex evoked by intense electrical stimulation of the median nerve (Hand-Blink Reflex; HBR), has been extensively used to derive fine-grained maps of defensive peripersonal space. However, as other subcortical reflexes, the HBR might also be modulated by lower-level factors that do not bear direct relevance to the defensive value of blinking, thus posing methodological and interpretive problems. Here, we tested whether HBR magnitude is affected by the muscular effort present when holding the hand in certain postures. We found that HBR magnitude increases with muscular effort, an effect most likely mediated by the increased corticospinal drive. However, we found strong evidence that this effect is substantially smaller than the well-known effect of eye-hand proximity on HBR magnitude. Nonetheless, care should be taken in future experiments to avoid erroneous interpretations of the effects of muscular effort as indicators of behaviour relevance.

## Highlights

• The magnitude of hand-blink reflex is increased by tonic cortico-spinal activation.
• This effect is smaller than the commonly observed HBR increase when the stimulated hand is near the eye.
• Nonetheless, when using HBR as an indicator of behavioural relevance, this effect should be taken into account.

## 1 Introduction

The eye blink in response to intense environmental stimuli is a prototypical defensive response to potential threats to the body [1], and it is typically evoked by a variety of somatosensory and non-somatosensory stimuli. When elicited by intense somatosensory stimuli delivered to the median nerve, the blink reflex is referred to as the hand-blink reflex (HBR) [2].

After the first descriptions of the physiological properties of the HBR in clinical neurophysiology in the late ‘90 s [[2], [3], [4]], the recording of the HBR has recently gained traction to investigate the spatial properties of the brain’s representation of environmental threats [[5], [6], [7], [8]]. Indeed, despite being entirely mediated by a subcortical circuit, we have shown that the HBR magnitude is enhanced when the stimulated hand is located closer to the face [5]. Furthermore, the magnitude of the HBR at particular points in space is affected by contextual factors such as the estimated defensive value of physical barriers [5], the anxiety trait [9], gravitational cues [10], motion of the stimulated hand [11,12], interpersonal interactions [8], chronic pain conditions [13], and blindness [14]. Given that at least some of these factors reflect cortical processing, it is probable that these effects are enacted by a cortical modulation on the excitability of the brainstem circuits subserving the HBR [5,15], and thus represent behaviourally relevant modulations of a response aiming to minimise damage to the body [16].

By exploring the HBR modulation by a large number of spatial locations of the stimulated hand in egocentric coordinates, we have characterized the fine-grained spatial properties of what we labeled the defensive peripersonal space (DPPS) surrounding the face [5,6]. This has furthered the understanding of how the position of potentially noxious stimuli affects the relevance of defensive avoidance responses [16].

However, in some of these experiments we have anecdotally observed that the HBR magnitude appeared to be increased in postures that require considerable effort to be reached and maintained. This idea is consistent with the well-known facilitation of subcortical reflexes when organisms exert a muscular effort, such as during the Jendrassik manoeuver [17]. Therefore, it is possible that the HBR magnitude is partially determined by the tonic corticospinal drive required to place and keep the hand in a particular position. Clarifying this possibility is important, since such an increase in HBR magnitude would not reflect the cortex’s influence on a defensive response, making the reverse inference between HBR magnitude and defensive behavioural relevance of a stimulus less likely to be correct (see Box 1 in [18]).

Here, we empirically assessed whether such an effect of effort on the HBR exists.

## 2 Materials and method

### 2.1 Participants

We analysed data from 14 participants (9 women, mean age 24.8 ± 5.0 yrs) to test whether the effort required to keep the hand in certain stimulation positions contributes to the HBR magnitude. To identify these HBR 'responders' [2,3,5], we collected data from 19 participants. Only those showing a HBR magnitude more than two standard deviations above pre-stimulus EMG activity in more than 40% of trials were considered to be HBR responders. Hence, 74% of subjects were HBR responders. This percentage is consistent with previous reports [5,9,15]. Participants gave written, informed consent before taking part in the study. The study was approved by the local ethics committee.

### 2.2 Stimulation

Participants were seated in a comfortable chair. Somatosensory stimuli consisted of constant-current squared pulses generated by an electrical stimulator (DS7A, Digitimer). Stimuli were delivered using a surface bipolar electrode placed on the right median nerve at the wrist. Stimulus duration was 200 μs, and the interval between successive stimuli was 30 s. In each participant, we first determined the stimulus intensity able to elicit a well-defined and stable blink reflex in response to electrical stimulation of the median nerve at the wrist (mean intensity 36.8 ± 11.3 mA). This was achieved by increasing the stimulus intensity until a clear HBR was observed in three consecutive trials, or the participant refused a further increase of stimulus intensity [2,3].

EMG activity was recorded from the orbicularis oculi muscle bilaterally, using pairs of surface electrodes with the active electrode over the lower eyelid and the reference electrode a few centimeters laterally to the outer canthus. Signals were amplified and digitized at a sampling rate of 8,192 Hz, and stored for off-line analysis.

### 2.3 Experimental Procedures

**Fig. 1:**
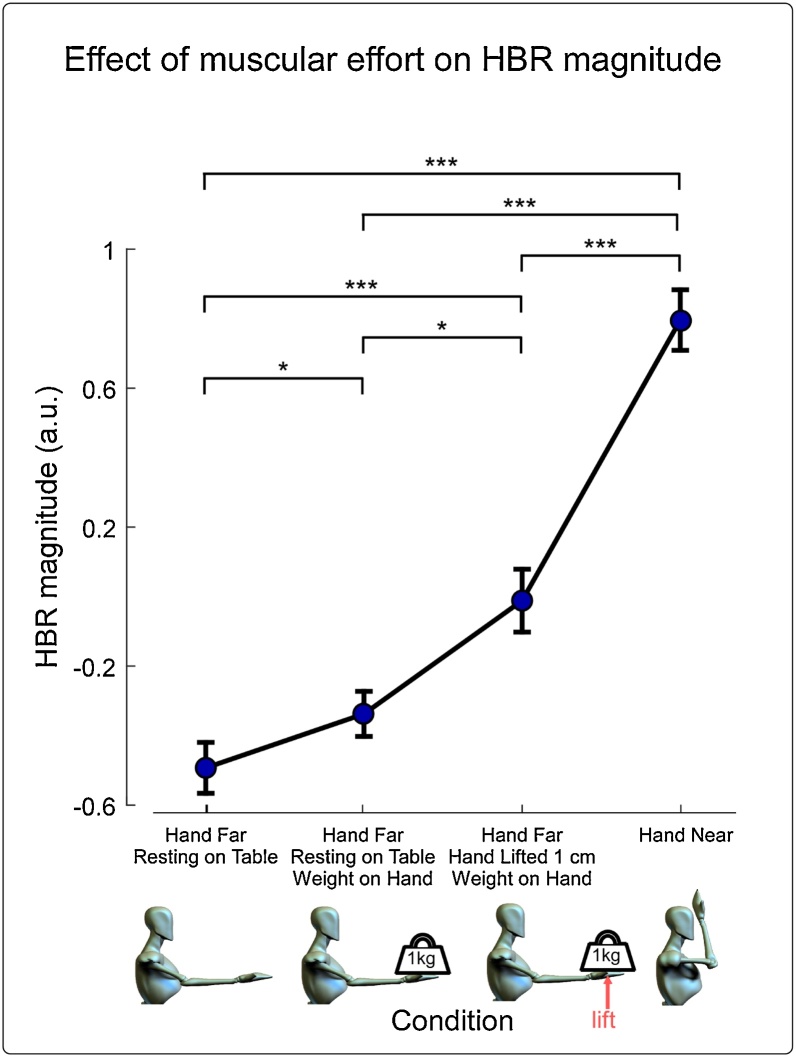
**Contribution of muscular effort to HBR magnitude.** The HBR was elicited by somatosensory stimulation of the right hand, and was recorded through surface electrodes placed beside and underneath each eye, in four conditions. In Condition 1, the hand was resting on a desk in front of the participant with the palm facing upwards (position ‘Far’, ∼60 cm from the face). In Condition 2, the hand was in the same ‘Far’ position, but a 1 kg weight was placed on the palm. Condition 3 was identical to Condition 2 (hand in position ‘Far’, with a weight on it), but participants were instructed to raise their entire forearm ∼1 cm off the desk whilst holding the weight – a posture requiring considerable effort to keep the hand in position. In Condition 4, the hand was in the ‘Near’ position, i.e. at eye-height, ∼4 cm in front of the face. Error bars display the standard error of the mean. Evidence of statistical difference between HBR magnitudes elicited with the hand in different positions is indicated by asterisks: *** p < 0.001, ** p < 0.01, * p < 0.05. The results confirm the hypothesis that an increase in tonic muscular effort results in an increase of HBR magnitude (Condition 1/2 vs Condition 3). However, the increase in HBR magnitude due to the proximity effect (Condition 1 vs Condition 4) is larger than that due to effort.

Somatosensory stimuli were delivered in four different conditions (as in Fig. 1, below). In Condition 1, the hand was resting on a desk in front of the participant with the palm facing upwards (position ‘Far’, approximately 60 cm from the face). In Condition 2, the hand was in the exact same ‘Far’ position, but a 1 kg weight was placed on the palm. Condition 3 was identical to Condition 2 (hand in position ‘Far’, with a weight on it), but participants were instructed to raise their entire forearm approximately 1 cm off the desk whilst holding the weight – thus requiring considerable effort to keep the hand in position. In Condition 4 the hand was in the ‘Near’ position, i.e. at eye-height, approximately 4 cm in front of the face.

We assumed the effort required to hold the hand in a given position is roughly proportional to the moment of force of the arm on the shoulder. We also assumed that the arm weighs 4 kg, has equal radius and density along its length, and hence that the weight of the arm acts at its centre of mass. We finally assumed that the arm is 70 cm long, that it is completely extended in the Condition 1, and that the shoulder is 10 cm behind the face. Therefore, the moment of force at the shoulder in Conditions 1 and 2 is approximately 0 Nm, because the arm is resting on the table (although the muscular effort in Condition 2 is probably slightly higher than in Condition 1 due to the need to stabilise and hold on to the 1 kg weight). In Condition 3, the moment of force is approximately 21 Nm (9.81 N/kg x (0.35 m x 4 kg + 0.7 m x 1 kg)). In Condition 4, the moment of force is approximately 3 Nm (0.07 m x 4 kg x 9.81 N/kg).

### 2.4 Data analyses and statistics

EMG signals from each participant were high-pass filtered (55 Hz) and full-wave rectified. The HBR magnitude was calculated as the area-under-curve (AUC) of each single-trial response, separately for each recording site. AUCs were first averaged across ipsilateral and contralateral recording sites and then across trials within each Condition. Finally, AUCs were normalized for each subject as Z-scores.

To investigate whether there was a difference in HBR magnitude between conditions, we performed a one-way repeated-measures ANOVA with four levels – one for each condition. We performed Mauchly’s test of sphericity to check whether corrections should be made to the results. We subsequently performed false-detection-rate corrected post-hoc paired t-tests between all conditions in order to identify any source of difference in HBR magnitudes.

## 3 Results

The repeated measures ANOVA showed strong evidence for a difference in HBR magnitudes between experimental conditions (F = 92.756, p = 2.62*10^−4^, Sphericity not violated; p = 0.0786). Post-hoc, FDR-corrected, paired t-tests between conditions showed strong evidence that the HBR magnitude was larger in Condition 4 (i.e. when the hand was near the face) than in all other three Conditions (i.e. when the hand was far from the face) (Condition 1 vs Condition 4: p = 6.68*10^-8^, Cohen’s d = 6.23; Condition 2 vs Condition 4: p = 1.49*10^-7^, Cohen’s d = 5.79; Condition 3 vs Condition 4: p = 6.26*10^-5^, Cohen’s d = 3.20). Importantly, there was also strong evidence that HBR magnitude was larger in Condition 3 (i.e. hand far from face during effort) than in Condition 1 (i.e. hand far from face without effort) (p = 5.07*10^-5^, Cohen’s d = 2.52). There was also evidence that HBR magnitude was larger in Condition 3 (i.e. hand far from face during effort) than in Condition 2 (i.e. hand far from face, with a weight resting on the hand) (p = 0.0214, Cohen’s d = 1.41), and weak evidence that it was larger in Condition 2 (i.e. hand far from face, with a weight resting on the hand) than in Condition 1 (i.e. hand far from face) (p = 0.0452, Cohen’s d = 1.20).

## 4 Discussion

These results indicate that during muscular effort the HBR is increased in magnitude. This effect is most likely consequent to the tonic activation of the corticospinal tract subserving the force needed to hold the weight of 1 kg with the hand receiving the somatosensory stimulus. The alternative possibility that the effect is consequent to the additional somatosensory input represented by the weight on the palm of the hand is not only physiologically less plausible [17] but also ruled out by the evidence that the HBR in Condition 3 - when the subject had to lift the weight - was larger than in Condition 2 - when the subject has the weight resting on the hand, but does not have to lift it.

The implications of this observation for empirical studies depend on a number of factors, which we discuss below. First, it is important to note that the size of HBR increase due to muscular effort (exemplified by comparing Condition 3 to Condition 2), is substantially smaller than the main 'Far-Near' effect of hand position (exemplified by comparing Condition 4 to Condition 1). Second, the muscular effort required to lift a weight at arm’s length (Condition 3; roughly 21 Nm, see Methods) is substantially larger than the muscular effort required to place and hold the hand close to the face (Condition 2; roughly 3 Nm, see Methods) (Fig. 1). Under the reasonable assumption that the facilitation exerted by the tonic activation of the corticospinal tract on HBR excitability is monotonically related to the corticospinal firing, the possibility that muscular effort explained previous changes of HBR magnitude due to differences in hand positions is minor. An alternative but less likely possibility is that the effect of effort and the effect of position interact with each other, resulting, in certain hand positions, in a much larger interaction effect of effort. This possibility remains to be explored.

Thus, the presence of an effect of muscular effort, however small it may be, raises the issue of how much this effect has contributed to the results of previously published experiments. A brief discussion of studies which might have been confounded by the effects of effort is warranted. For example, Wallwork et al. [11] found that the act of moving the hand towards the face increased the HBR magnitude while the hand was far from the face. One might imagine that the increased effort necessary to lift and move the arm towards the face could have contributed to the observed effect. However, in a very similar experiment requiring identical movement of the arm, this effect was never observed [12]. The difference in results between the two experiments is thus likely driven by differences in stimulus presentation, which resulted in differences in cognitive expectation between the two experiments, as discussed in detail in [19]: in one Experiment stimuli were predictable [11], while in the other they were not [12]. Thus, it is likely that simply moving the arm does not constitute a large enough increase in effort to cause a measurable increase in HBR magnitude.

Another set of experiments possibly influenced by effort explores the magnitude of HBR as a function of the hand across horizontal and vertical directions [6]. In these experiments, the HBR magnitude was found to be larger when the hand was held above the face than when the hand was held equally distant beneath the face. This result was explained as an effect of gravity on the brain’s estimate of the probability that the threat (represented by the somatosensory stimulus eliciting the reflex) will harm the body: when stimuli are above the body, they are more likely to move downwards to interact with, and thus harm the body. A follow up experiment tested this interpretation explicitly, and, importantly, for the first time controlled for the effect of effort by having experimenters hold the participants’ arms in place [10]. In doing so, the participants’ muscular effort necessary for holding each of the used hand postures was at least substantially reduced. While this study still revealed an increased HBR magnitude when the stimulated hand was above eye level as compared to when it was at the same distance below eye level, the increase was smaller [10]. Thus, the discrepancy in size of the effect of gravity might in fact be due to effort. As is often the case however, other explanations cannot be excluded. For example, the cortex might rely on muscular activation in order to effectively update its estimate of the hand’s position. If this were the case, the decrease in effort caused by experimenters holding participants’ arms might have coincided with a decrease in proprioceptive awareness, which in turn resulted in the observed difference. This would mean that the brain might use muscular activation as a gravitational cue when it updates the DPPS. Of course, Occam’s Razor makes this option the least likely.

Thus, the reported results indicate that future HBR experiments should be designed taking this issue into account. For example, the effort required to hold the arm raised and outstretched is greater than that required to hold it raised but near the body, and so the effect of effort scales inversely with distance. Therefore, if one were to compare the HBR proximity effect (as done in [9]) between when the arm is lifted by the participant and when the arm is held in place by the experimenter, one might erroneously conclude that the defensive field mapped out by the HBR has changed. Hence, while the effect of effort is relatively small, experiments exploiting small differences in HBR magnitude should control for it, either by supporting the arm, or by matching effort across tested conditions.

In more general terms, these results can serve as a reminder that even responses which are very reliably modulated by high level factors, and therefore clearly seem to have a particular interpretation, can still be modulated by confounding factors and lead to erroneous conclusions. Reverse inference, thus, remains a dangerous game [18].
